# Characterization of hotspot exonuclease domain mutations in the DNA polymerase ϵ gene in endometrial cancer

**DOI:** 10.3389/fonc.2022.1018034

**Published:** 2022-10-12

**Authors:** Wenjuan Tian, Zhaodong Ji, Jingshu Wang, Jiao Meng, Rui Bi, Yulan Ren, Boer Shan, Gong Yang, Huaying Wang

**Affiliations:** ^1^ Department of Gynecologic Oncology, Fudan University Shanghai Cancer Center, Shanghai, China; ^2^ Department of Oncology, Shanghai Medical College, Fudan University, Shanghai, China; ^3^ Department of Clinical Laboratory, Huashan Hospital, Fudan University, Shanghai, China; ^4^ Central Laboratory, The Fifth People’s Hospital of Shanghai, Fudan University, Shanghai, China; ^5^ Cancer Institute, Fudan University Shanghai Cancer Center, Shanghai, China; ^6^ Department of Pathology, Fudan University Shanghai Cancer Center, Shanghai, China

**Keywords:** endometrial cancer, DNA polymerase epsilon, 3’-5’-Exonuclease, DNA replication, hotspot exonuclease domain mutations

## Abstract

**Objective:**

This study was aimed to profile hotspot exonuclease domain mutations (EDMs) of the DNA polymerase ϵ gene (*POLE*) in endometrial cancer (EC) and to investigate the effects of EDMs on tumor cell behavior and catalytic activities of Polϵ.

**Methods:**

*POLE* sequencing was performed in tumor tissue samples from patients with EC to identify hotspot EDMs. Bioinformatics tools were used to select the potential pathogenic EDMs. The association of EDMs with the clinical outcomes of patients was assessed. EC cells were transfected with wildtype *POLE* or *POLE* variants to examine the effects of the EDMs on EC cell behavior, including cell cycle, migration, and invasion. Co-immunoprecipitation was employed to obtain FLAG-tagged wildtype and mutant catalytic subunits of Polϵ, followed by the assessment of polymerase and exonuclease activities.

**Results:**

In addition to previously reported P286R and V411L, R375Q and P452L were identified as novel, and deleterious *POLE* hotspot EDMs of EC. Patients in EDM group had significantly better clinical outcomes than the rest of the cohort. Compared with wildtype POLE, overexpression of POLE variants promoted cisplatin resistance, G0/G1 cell cycle arrest, and cell migration and invasion in EC cells. Overexpression of POLE variants significantly increased the abundance of 3’-OH and upregulated the expression of DNA mismatch repair genes in HEK293T cells. Compared with wildtype Polϵ, Pol ϵ mutants exhibited undermined polymerase and exonuclease abilities in the presence of mismatched nucleotides in HEK293 cells.

**Conclusion:**

We characterized the of hotspot exonuclease domain mutations in the DNA polymerase ϵ gene and identified P286R, V411L, R375Q, and P452L as pathogenic *POLE* hotspot EDMs in endometrial cancer. These hotspot EDMs are associated with the malignant behavior of endometrial cancer cells *in vitro* and favorable prognosis in patients, suggesting that *POLE* affects a wide range of cellular processes beyond DNA replication and proofreading.

## Introduction

Endometrial cancer is the sixth most common cancer in women worldwide. It is estimated that the incidence rate of endometrial cancer will increase by more than 50% by 2040 (1). The DNA polymerase ϵ gene (*POLE*)-encoded Polϵ is one of the three DNA polymerases that are required for eukaryotic genome replication. The 140 kDa N-terminal catalytic subunit of Polϵ harbors 5’–3’ polymerase and 3’–5’ exonuclease activities, playing critical roles in DNA replication and proofreading in the newly synthesized DNA strand (2). Somatic *POLE* exonuclease domain mutations (EDMs) occur in 7–12% of endometrial cancer cases, with P286R and V411L representing the most common pathogenic *POLE* EDMs, followed by S297F, A456P, and S459F (3). It has been suggested that somatic *POLE* EDMs are early events in endometrial cancer because the presence of a *POLE* mutation leads to an extremely high mutation load in precancerous lesions and thus accelerates their transition to cancer (4, 5). However, less frequent *POLE* variants that might also contribute to the pathogenesis of endometrial cancer remain largely unknown.

Recently, whole‐genome sequencing or targeted *POLE* sequencing has been widely used to identify new *POLE* EDMs involved in endometrial cancer (6). Considering the difference between driver mutations that push a cell toward a cancerous state and passenger mutations that are not directly responsible for the cancer phenotype of the cell, it is critical to characterize the pathogenicity of newly identified *POLE* EDMs (7). Bioinformatics tools, such as MutationTaster, SIFT, AlignGVGD, and Polyphen, are commonly used to distinguish driver mutations from passenger mutations (8–11). Studies have shown that pathogenic *POLE* EDMs are associated with excellent prognosis in patients with endometrial cancer due to enhanced antitumor immune response (12, 13). Polϵ has also been linked to cell cycle checkpoint activation in human cells (14). These findings suggest that beyond replication fidelity, *POLE* EDMs may affect a wider range of cellular processes. Thus, the associations of newly identified EDMs with the clinical outcomes and tumor cell behavior should also be considered when interpreting the *POLE* mutations in endometrial cancer (15).

In this study, by targeted sequencing of *POLE* in tumor tissue samples from 138 patients with endometrial cancer, we identified four *POLE* hotspot EDMs P286R, V411L, R375Q, and P452L, among which R375Q and P452L were reported for the first time. To characterize the pathogenicity of the EDMs, we investigated the associations of *POLE* variants with the clinical outcomes of the patients, their influences in chemosensitivity, cell cycle transition, and metastatic abilities of endometrial cancer cell lines, as well as the polymerase and exonuclease activities of the recombinant Polϵ mutants. Our results suggest that P286R, V411L, R375Q, and P452L are pathogenic *POLE* hotspot EDMs in endometrial cancer and affect the malignant behavior of tumor cells, which may provide new insights into the pathogenesis of endometrial cancer.

## Results

### *POLE* hotspot EDMs are associated with favorable clinical outcomes in patients with endometrial cancer

**Figure 1:**
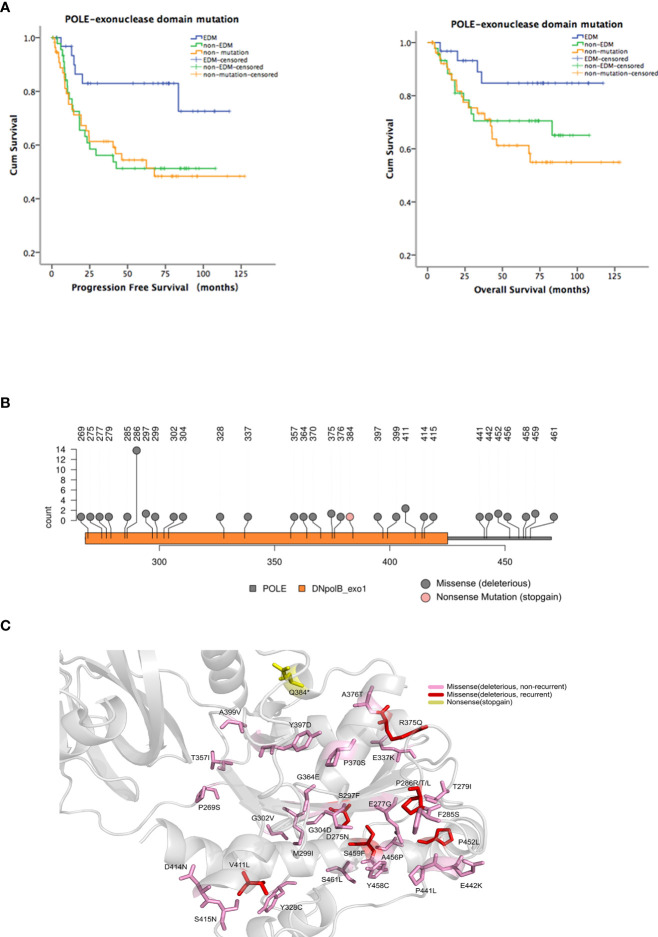
Identification of hotspot exonuclease domain mutations (EDMs) in the DNA polymerase ϵ gene (*POLE*) in endometrial cancer. A total of 35 nonsense/stop-gain, 39 frameshift, and 57 missense mutations were identified in 69/138 (50%) endometrial tumor samples from patients. Among these mutations, there were 49 *POLE* EDMs, comprising 1 nonsense/stop-gain mutation and 48 missense mutations, in 32/138 (23.2%) samples. **(A)** Patients were divided into EDM (n = 32), non-EDM (n = 37), and non-mutation (n = 69) groups. Kaplan-Meier analysis was performed to evaluate the association of *POLE* EDMs with progression free survival (PFS) and overall survival (OS). Blue line: patients with *POLE* EDM tumors; green line: patients with non-EDM tumors; orange line: patients with non-mutation tumors. **(B)** Prediction of deleterious EDMs. Pink dot: stop mutations; gray dot: deleterious mutations. **(C)** Modelling of the exonuclease domain mutations in POLE. Gray, 9–14 exons, 268–471 residues; pink, unrepeated deleterious mutations; red, recurrent deleterious mutations; yellow, stop mutations.

To explore the presence of *POLE* variants and their association with clinical outcomes in endometrial cancer, we retrospectively analyzed the tumor samples and clinical characteristics of 146 patients with endometrial cancer. After exclusion of control failed 8 samples that failed quality control, we obtained the *POLE* sequencing results of 138 samples. In this cohort, we identified 35 nonsense/stop-gain, 39 frameshift, and 57 missense mutations in 69/138 (50%) samples. Among these mutations, there were 49 *POLE* EDMs, comprising 1 nonsense/stop-gain mutation and 48 missense mutations, in 32/138 (23.2%) samples. To analyze the association of the EDMs with clinical outcomes, we divided the patients into EDM (n = 32), non-EDM (n = 37), and non-mutation (n = 69) groups and performed Kaplan-Meier analysis. The EDM group had a significantly better PFS than the non-EDM (82.9% vs. 51.3%, *P* = 0.019) and non-mutation groups (82.9% vs. 54.4%, *P* = 0.01) and a significantly better OS than the non-mutation groups (84.7% vs. 61.2%, P = 0.019) (**Figure 1A**). No significant difference of OS was observed between the EDM and non-EDM groups (84.7% vs. 70.5%, P = 0.103). These results suggest that the EDM group had a better prognosis compared with the rest of the cohort.

The most common EDMs identified in this study were P286R/T/L (n = 13), V411L (n = 3), S297F (n = 2), G304D (n = 2), R375Q (n = 2), P452L (n = 2), and S459F (n = 2). Among them, P286R, V411L, S297F, and S459F had been reported as the most common *POLE* mutations, and the pathogenicity was confirmed [15, 18]. R375Q and P452L were newly identified and predicted as deleterious mutations by SIFT, PolyPhen-2, and AlignGVGD (**Figures 1B, C**). Therefore, P286R, V411L, R375Q, and P452L were designated as hotspot EDMs and were further characterized and assessed for the pathogenicity in endometrial cancer.

### POLE EDMs induce cisplatin resistance in endometrial cancer cells

**Figure 2:**
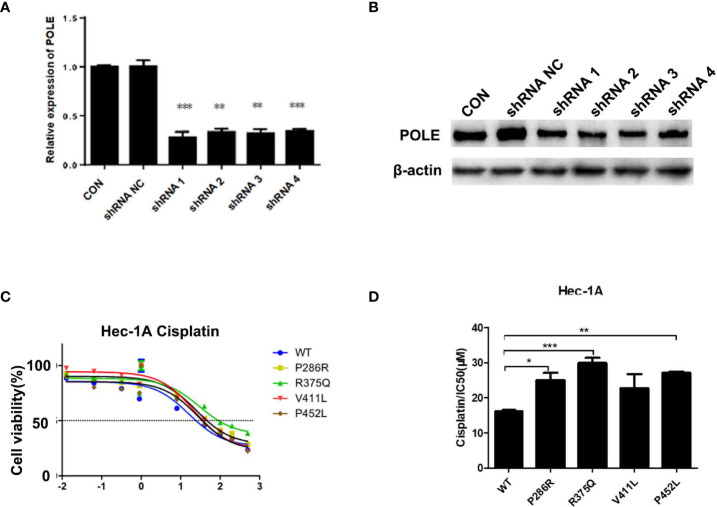
*POLE* EDMs induced cisplatin resistance in endometrial cancer cells. **(A, B)** HEK-293T cells were transfected with lentiviral vectors expressing negative control small hairpin RNA (shRNA NC) or shRNAs against *POLE* (shRNA1, shRNA2, shRNA3, or shRNA4). Quantitative real-time PCR (qRT-PCR) and Western blot analysis were performed at 48 h after transfection to determine mRNA and protein levels of POLE. **(C, D)** HEC-1A cells were transiently transfected with lentiviral vectors expressing wildtype *POLE* or P286R, R375Q, V411L, or P452L variant. Cells were treated with different concentrations of cisplatin (0.0001, 0.0128, 0.064, 0.32, 1.6, 8, 40, 100, 200, and 500 µmol/L) for 48–72 (h) MTT assay was conducted to measure the cell viability. IC50 was calculated. Data are expressed as the mean ± standard deviation (SD). *P < 0.05, **P < 0.01, ***P < 0.001, vs. WT; n = 3. WT, wildtype.

To characterize the hotspot EDMs, we stably silenced POLE in HEK-293T cells. HEK293T cells were used to optimize the knockdown of POLE (**Figures 2A, B**). HEC-1A and AN3CA cells that exhibited dramatically weaker POLE protein expression than other endometrial cancer cell lines (**Supplementary Figure 2**) were selected for the following experiments.

To explore whether the good prognosis in the EDM group is associated with chemosensitivity, we overexpressed wildtype POLE or POLE variants in endometrial cancer cells and examined the response to different doses of cisplatin. By MTT assay, we found that overexpression of POLE-P286R, R375Q, and P452L significantly increased IC_50_ values of cisplatin compared with overexpression of wildtype POLE (**Figures 2C, D**), suggesting that the hotspot mutations induce cisplatin resistance in endometrial cancer. Thus, it appears that the good prognosis of the EDM group may not be not associated with increased chemosensitivity.

### *POLE* EDMs induce G0/G1 cell cycle arrest in endometrial cancer cells

**Figure 3:**
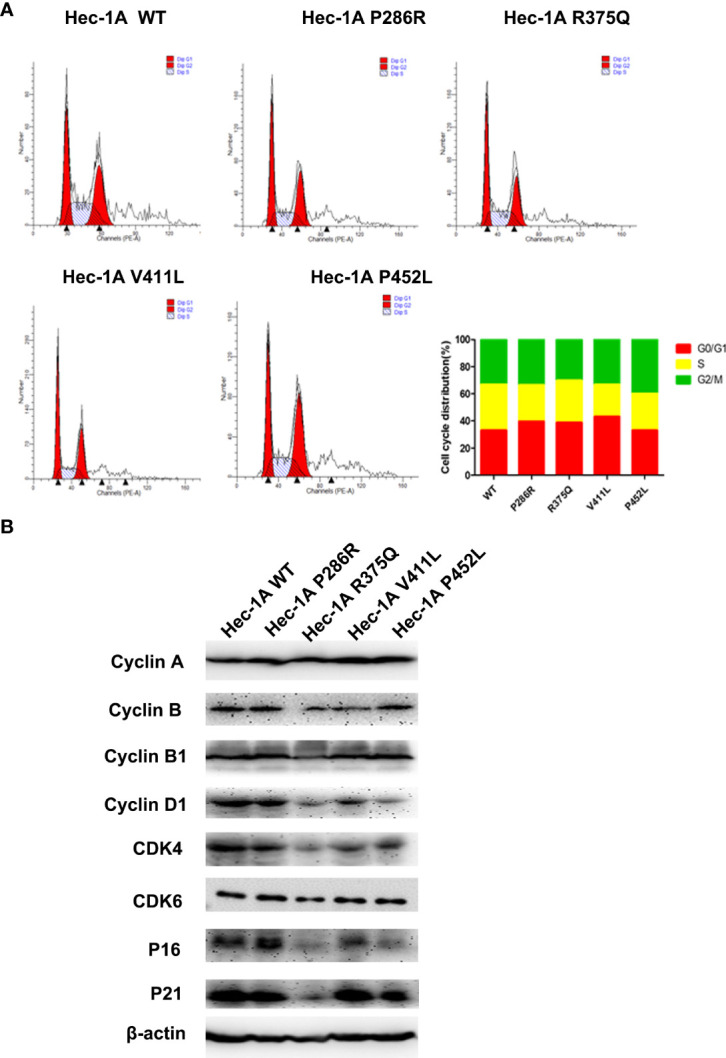
*POLE* EDMs induced G0/G1 cell cycle arrest in endometrial cancer cells. HEC-1A cells were transiently transfected with wildtype *POLE* or P286R, R375Q, V411L, or P452L variant. **(A)** Flow cytometry analysis was conducted at 48 h after transfection to analyze the cell cycle. **(B)** Western blot analysis was carried out at 48 h after transfection to measure the protein levels of Cyclin A, clyclin B, cyclin B1, cyclin D1, CDK4, CDK6, P16, and P21. β-actin was used as an internal reference. Data are expressed as the mean ± SD.

Then, we explored the effects of the hotspot mutations on cell cycle by flow cytometry.The data analysis showed that overexpression of *POLE* variants resulted in increases in the proportions of G0/G1-phase cells (6% for P286R, 6% for R375Q, and 10% for V411L) and decreases in the proportions of S-phase cells (7% for P286R, 3% for R375Q, 10% for V411L, and 7% for P452L), compared with overexpression of the wildtype *POLE* (**Figure 3A**). We also observed the reduced expression of G1/S transition-related proteins including Cyclin D1, CDK4, CDK6, and P16 (**Figure 3B**). These results suggest that *POLE* hotspot EDMs promote G0/G1 cell cycle arrest in endometrial cancer cells.

### *POLE* EDMs promote cell migration and invasion of endometrial cancer cells

**Figure 4:**
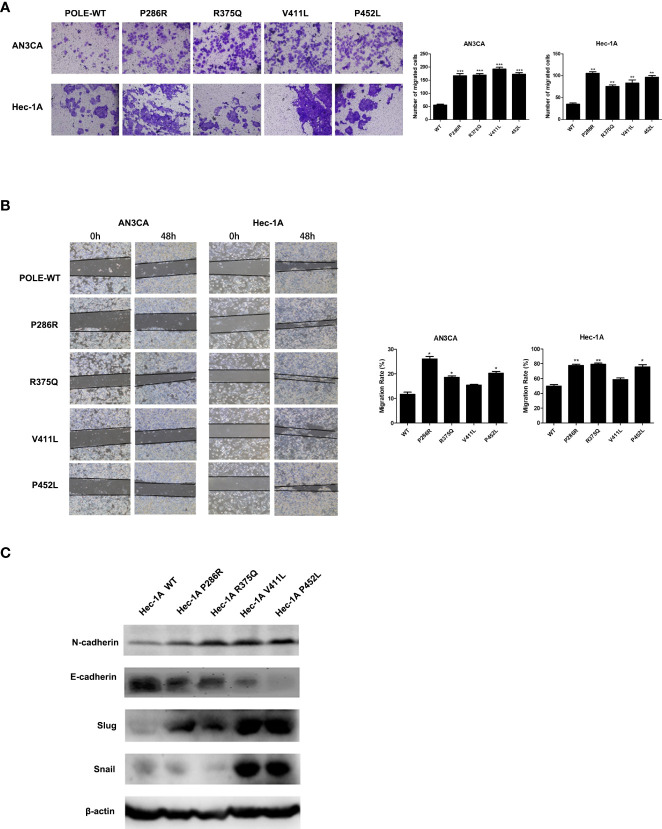
*POLE* EDMs promoted cell migration and invasion of endometrial cancer cells. HEC-1A cells were transiently transfected with wildtype *POLE* or P286R, R375Q, V411L, or P452L variant. **(A)** Transwell analysis was performed to examine cell invasion. **(B)** Wound healing assay was conducted to examine cell migration. **(C)** Western blot was carried out at 48 h after transfection to measure the protein levels of N-cadherin, E-cadherin, Slug, and Snail. β-actin was used as an internal reference. Data are expressed as the mean ± SD. *P < 0.05, **P < 0.01, ***P < 0.001, vs. WT; n = 3. WT, wildtype.

To explore the effects of *POLE* hotspot EDMs on cell metastatic abilities, we performed Transwell assay in HEC-1A and AN3CA cells. Compared with wildtype *POLE*, overexpression of *POLE* variants remarkably promoted the invasiveness abilities of both cell lines. Except for *POLE*-V411L, the other variants also significantly enhanced the migrating abilities of both cell lines (**Figures 4A, B**). Western blot analysis revealed that all variants enhanced protein expression of N-cadherin and slug while attenuating protein expression of E-cadherin in HEC-1A cells compared with wildtype *POLE* (**Figure 4C**). We also observed dramatic upregulation of snail in *POLE*-V411L- and *POLE*-P425L-overexpressing cells. Taken together, these findings suggest that *POLE* hotspot EDMs might contribute to epithelial-mesenchymal transition to promote endometrial cancer metastasis.

### *POLE* EDMs impair DNA mismatch repair in HEK293T cells

**Figure 5:**
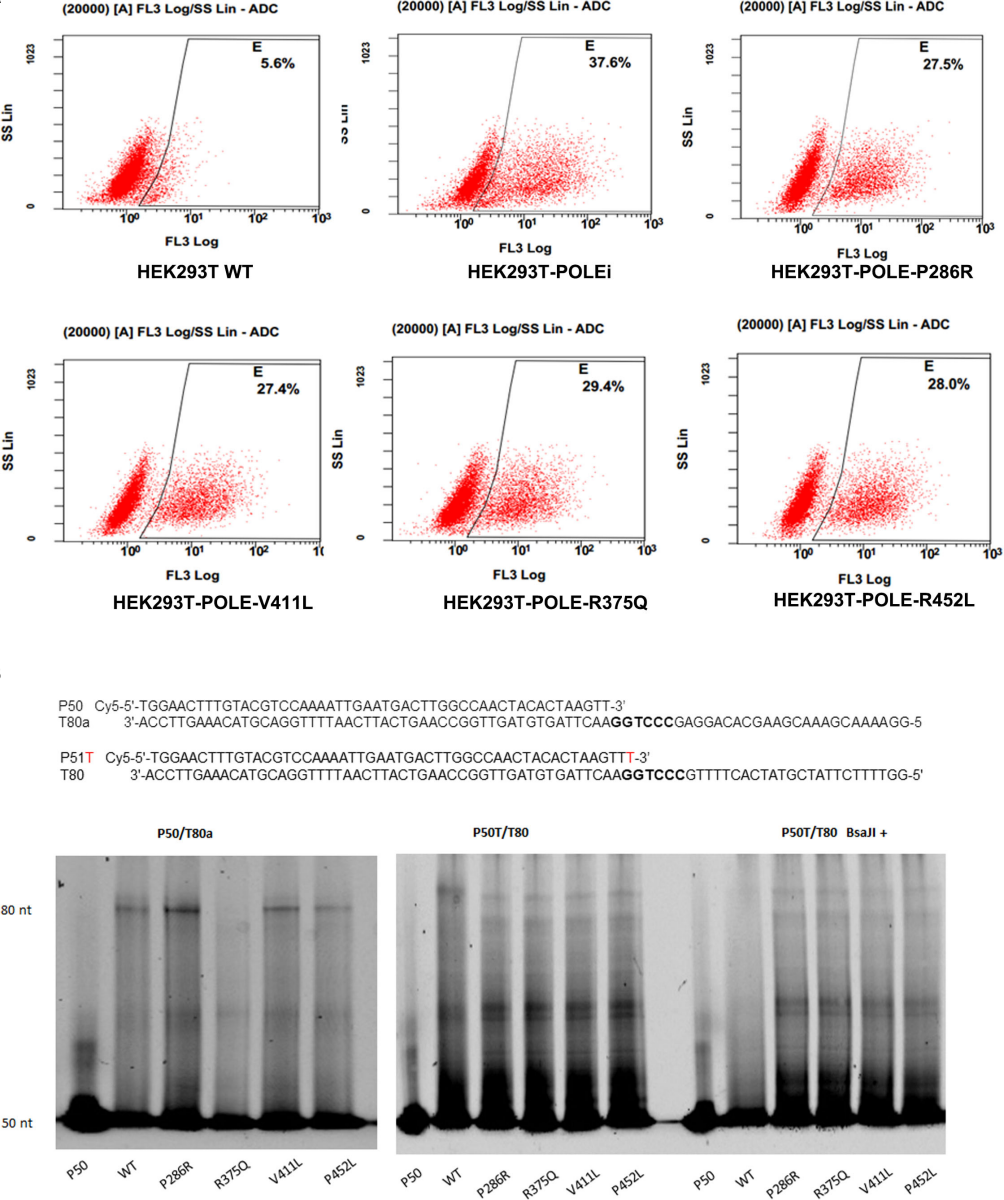
*POLE* EDMs impaired DNA mismatch repair in HEK293T cells. **(A)** HEK293T Cells were transfected with shPOLE, wildtype *POLE*, or *POLE* variants for 48 h, followed by fixing with formaldehyde and incubation with T7 endonuclease 1 at 37˚C for 45 min to cleave the mismatched base pairs. The produced 3′-OH DNA ends were stained with TUNEL. Flow cytometry analysis was performed to quantify the mismatched base pairs. **(B)** Wildtype Polϵ or Polϵ mutants were incubated with the P50/T80a (left panel) or P51T/T80 (middle panel) substrate at 30 °C for 30 min. Right panel: Wildtype Polϵ or Polϵ mutants were incubated with P51T/T80 at 30 °C for 30 min, followed by *BsaJI* digestion. Urea polyacrylamide gel electrophoresis was performed to examine the polymerase and exonuclease capacities.

Because the exonuclease domain of Polε carries a 3′–5′ proofreading activity that removes misincorporated nucleotides, we sought to investigate whether *POLE* EDMs affect DNA mismatch repair. The T7 endonuclease I cleavage assay showed that knockdown of *POLE* or overexpression of the *POLE* variants resulted in increased abundance of 3’-OH in HEK293T cells, compared with overexpression of wildtype *POLE* (shPOLE: 37.6% vs. 5.6%, P286R: 27.5% vs. 5.6%, V411L: 27.4% vs. 5.6%, R375Q: 29.4% vs. 5.6%, P452L: 28.0% vs. 5.6%; all *P* < 0.05; **Figure 5A**), suggesting that the hotspot EDMs are pathogenic mutations that compromise DNA proofreading in the cells.

### EDMs impair the polymerase and exonuclease activities of Polε

We next tested whether the hotspot mutations affect the polymerase and exonuclease activities of Polϵ. The 140 KDa FLAG-tagged wildtype and mutant catalytic fragments of human Polϵ were expressed in HEK293T cells and confirmed by Coomassie blue staining and Western blot analysis (**Supplementary Figures 2A, B)**. We incubated the purified recombinant proteins with correctly matched P50/T80a substrate or P51/T80 substrate containing a mismatched primer terminus and a *BsaJI* recognition sequence at the primer-template junction, respectively. In a primer extension assay using P50/T80a substrate, we found that like wildtype Polϵ, Polϵ-P286R, Polϵ-V411L, and Polϵ-P452L still retained polymerase activity, as evidenced by the accumulation of full-length products at 80-nt. Polϵ-P286R and Polϵ-V411L even exhibited significantly greater polymerase activities than wildtype Polϵ (**Figure 5B**, left panel). However, when incubated with the P51T/T80 substrate, all Polϵ mutants failed to produce the full-length products and only generated short sections of DNA (**Figure 5B**, middle panel). This finding suggests that the polymerase activity of Polϵmay be undermined by the hotspot mutations in the presence of mismatch nucleotides. Furthermore, after *BsaJI* digestion, only 51-nt fragment was present in the extension product generated by wildtype Polϵ, indicating that wildtype Polϵ excised the mismatched nucleotide (**Figure 5B**, right panel). However, the fragments resistant to *BsaJI* digestion were still present in the extension products generated by the Polϵ mutants, suggesting that the Polϵ mutants are unable to correct the mismatch due to the loss of exonuclease activity. Taken together, these results suggest that the hotspot EDMs are pathogenic mutations that reduce the mismatch extension and proofreading abilities of Polϵ.

**Figure 6:**
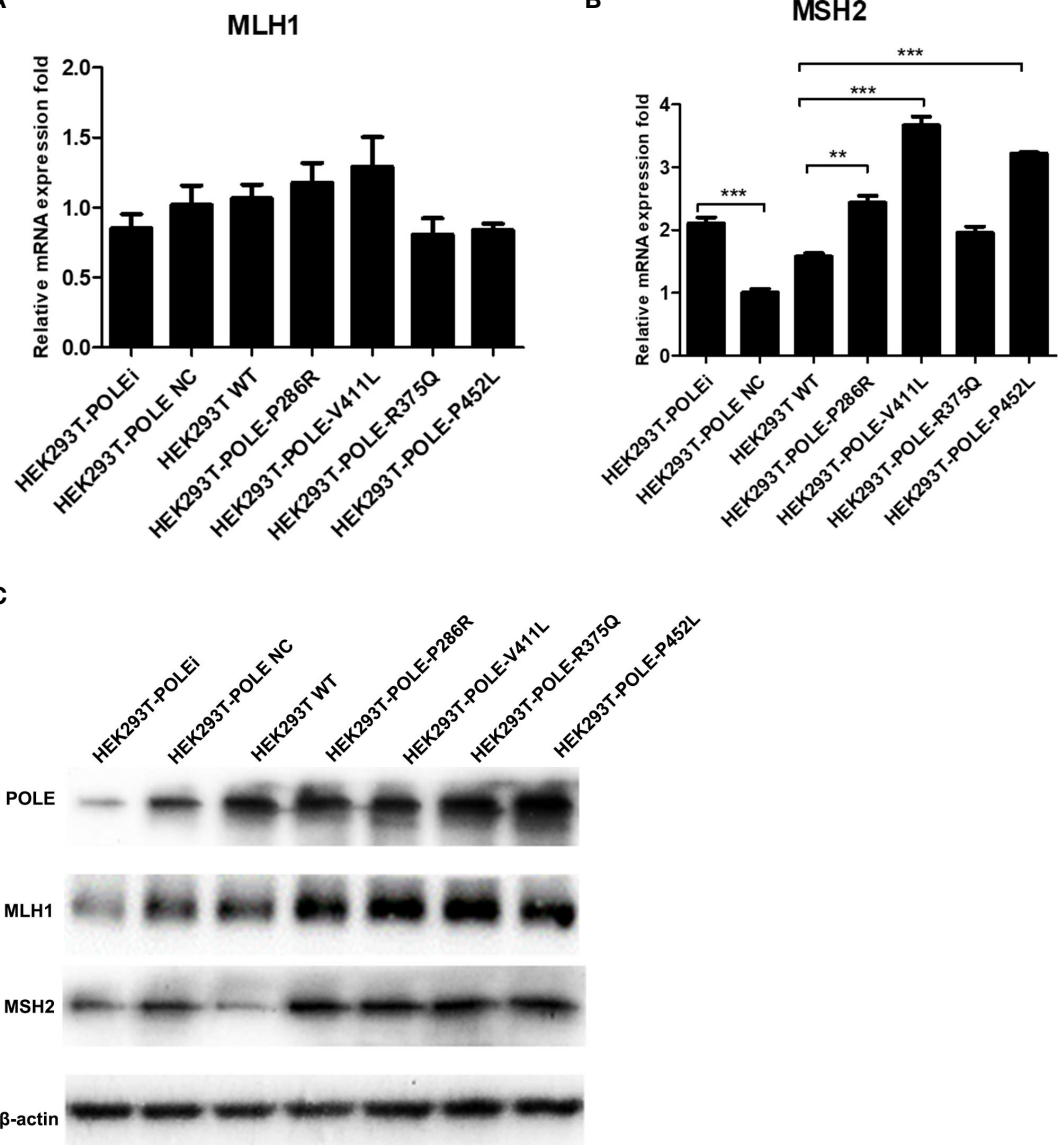
*POLE* EDM upregulated the expression of DNA mismatch repair genes. The stable *POLE*-knockdown HEK293T cells were transiently transfected with lentiviral vectors expressing wildtype *POLE*, *POLE* variants, or negative control. qRT-PCR **(A, B)** and Western blot analysis **(C)** were performed to measure mRNA and protein levels of POLE, MLH1, and MSH2. **P<0.01, ***P<0.001.

We also observed that compared with wildtype *POLE*, overexpression of *POLE* variants generally upregulated mRNA and protein expression of DNA mismatch repair genes MLH1 and MSH2 to different extent (**Figure 6**), suggesting that *POLE* EDM-induced DNA mismatch activates the mismatch repair system.

## Discussion

Endometrial cancer subtypes HGCC, SC, and CCC exhibit higher proportions of metastases and recurrences as well as poorer 5-year survival rates than low-grade endometrial endometrioid carcinomas (16). In this study, we performed targeted *POLE* sequencing in the tumor tissue samples of these three types of endometrial cancer and found that 32/138 (23.2%) of patients carried *POLE* EDMs. Kaplan-Meier analysis showed that patients with EDMs had significantly better PFS and OS than the rest of the cohort, suggesting a complex relationship between *POLE* mutations and the pathogenesis of endometrial cancer. To reveal the molecular mechanism underlying the role of *POLE* mutations in endometrial cancer, we selected four hotspot EDMs, including P286R, V411L, R375Q, and P452L, and assessed their pathogenicity based on the effects on catalytic activities of Polε and tumor cell behavior.

It has been suggested that *POLE* mutation impairs genomic stability by disabling proofreading. For example, Shinbrot et al. have shown that compared with wildtype *POLE*, the P286R variant essentially inactivates proofreading, whereas the V411L variant reduces exonuclease activity without completely abolishing it (17). Our results showed that Polϵ mutants lost the ability to correct the mismatched base pairs and generated the fragments that were resistant to *BsaJI* digestion, suggesting impaired proofreading activities in the mutants. Interestingly, in addition to disabling proofreading, *POLE* mutation also promotes ultramutation by stimulating polymerization. Xing et al. has found that the yeast Polϵ-P286R analog exhibits a dramatically increased DNA polymerase activity compared with wildtype Polϵ, leading to increased mismatch extension and bypass of hairpin DNA structure and therefore contributing to genomic mutation load (18). Similarly, our results showed that Polϵ-P286R and Polϵ-V411L exhibited significantly higher polymerase activities compared with wildtype Polϵ in the presence of a correctly matched substrate. However, we did not observe increased polymerase activity in the presence of a mismatched substrate. In addition, Polϵ-P452L still retained polymerase activity comparable to wildtype Polϵ, whereas Polϵ-R375Q barely showed any polymerase activity. These results suggest that *POLE* mutations have differential effects between yeast and human cells.

Regarding the role of POLE mutations in endometrial cancer, most studies are focused on the association of POLE mutations with clinical outcomes and antitumor immune responses, and only a few studies investigated the role of POLE mutations in chemoresistance (19, 20). To explore whether the favorable clinical outcomes of patients with POLE mutations are associated with diminished malignantlly, we examined chemoresistance, cell cycle transition, and cell migration and invasion of endometrial cancer cells in term of POLE mutations. We observed that POLE-P286R, R375Q, and P452L induced cisplatin chemoresistance in endometrial cancer cells. Consistent with our findings, Van Gool et al. demonstrated that POLE-P286R, S297F, and V411L mutations did not increase the sensitivity to chemotherapy or radiotherapy in mouse-derived embryonic stem cells (20). Similarly, Bellone et al. reported that the POLE-mutated endometrial carcinoma cells were resistant to carboplatin (19). Thus, the favorable prognosis of the EDM group is not secondary to the increased chemosensitivity but likely linked to the enhanced antitumor immune response (12, 13, 19).

It has been reported that high frequent POLE mutations are associated with metastatic tumors in synchronous endometrial and ovarian carcinoma (21). Imboden et al. found that POLE-mutated endometrial tumors proceed to lymph-node metastasis and present aggressive non-endometrioid subtypes (5). Our results showed that overexpression of *POLE* variants remarkably promoted the metastatic abilities and altered the expression of EMT markers of endometrial cancer cells compared with wildtype *POLE*, suggesting that POLE mutations might contribute to endometrial cancer metastasis. This may explain the results from other studies showing that POLE mutations are more frequently detected in high-grade endometrial tumors compared with low-grade tumors (22, 23). Unrepaired DNA damage in cell cycle can disrupt cell cycle progression and replication. Polϵ has been implicated in cell cycle checkpoint activation due to its essential role in DNA replication and proofreading (14). Our results show that *POLE* hotspot EDMs induce G0/G1 cell cycle arrest in endometrial cancer cells, which may contribute to the good prognoses of patients with EDMs.

In this study, in addition to the most common pathogenic *POLE* EDMs P286R and V411L, we identified two new hotspot DEMs R375Q and P452L in patients with aggressive endometrial cancer subtypes. We demonstrate that these hotspot EDMs are associated with favorable prognosis in patients. We also characterized these hotspot EDMs as pathogenic mutations, as evidenced by impaired polymerase and exonuclease activities of Polϵ resulting from the EDMs. Functionally, the hotspot EDMs promote chemoresistance, cell cycle arrest, and metastatic abilities of endometrial cancer cells *in vitro*, suggesting that *POLE* affects a wider range of cellular processes beyond DNA replication and proofreading.

## Materials and methods

### Patients and specimens

The candidates for this project were patients who have been diagnosed with endometrial cancer at Fudan University Shanghai Cancer Center (Shanghai, China) from January 2006 to December 2015, including 160 high-grade endometrial cancer (HGEC), 73 serous adenocarcinoma (SC), and 46 clear cell adenocarcinoma (CCC). Clinicopathological characteristics and paraffin-embedded cancer specimens of these patients were analyzed retrospectively. After excluding the patients with diagnostic errors, incomplete clinicopathological information, or low-quality/quantity cancer specimens, 88 HGEC, 29 SC, and 29 CCC tumor samples were eligible for inclusion. Progression-free survival (PFS) was the time from operation to recurrence or progression. The overall survival (OS) was the duration from operation to death or the last follow-up. This study was approved by the Institutional Ethics Committee of Fudan University Shanghai Cancer Center. A written consent was obtained from each patient.

### Cell lines and cell culture

Human embryonic kidney (HEK)293T cells and endometrial adenocarcinoma cell lines (HEC-1A and AN3CA) were purchased from the Cell Bank of Shanghai Institute of Biological Sciences, Chinese Academy of Sciences (Shanghai, China). HEK-293T, HEC-1A, and AN3CA cells were maintained in DMEM (Corning, Corning, NY, USA), McCoy’s 5A (Gibco, Thermo Fisher Scientific, Waltham, MA, USA), and RPMI 1640 (Corning) medium, respectively, supplemented with 10% fetal bovine serum (Gibco) and 1% ampicillin/streptomycin (Sangon Biotech, Shanghai, China) in a humidified atmosphere of 5% CO_2_ at 37 °C.

### *POLE* sequencing and bioinformatics analysis

Genomic DNA was extracted from paraffin-embedded tissue samples using a QIAamp DNA FFPE tissue kit (Qiagen, Hilden, Germany) following the manufacturer’s instruction. The DNA quality was analyzed using an Agilent 2100 bioanalyzer instrument (Agilent, Santa Clara, CA, USA). The primers for *POLE* sequencing were summarized in **Supplementary Table 1**. Sequencing was performed using the Illumina X-10 platform (Illumina, San Diego, CA, USA) with 2×150 bp paired-end reads.

Clean reads were obtained from raw reads by removing the adapters using Cutadapt (24) and were mapped to the human genome using the Burrows-Wheeler Alignment tool (25). Variants were called using the Genome Analysis Toolkit in the form of variant call format (26). A variant was considered valid when the overall read depth > 500 and the percentage of the variant in the overall read depth > 5%. The variants were annotated using ANNOVAR and aligned to GRCh38 (27). The pathogenicity of variants was predicted using SIFT, PolyPhen-2, and AlignGVGD, followed by evaluation using the ClinVar and InSiGHT databases. Data was analyzed and illustrated using python 2.7.15+R 3.5.1 and Bioconductor/trackViewer 1.18.2. The homologous structure of *POLE* was constructed using I-Tasser (28).

### Expression and purification of wildtype and mutant catalytic fragments of human Polϵ

The N-terminal half of *POLE* that encodes the DNA polymerase and exonuclease domains of human Polϵ (residues 1–1189, 140kDa) was cloned into a pHB-CMV-MCS-EF1-Puro vector as previously described (29). The plasmids encoding Polϵ-P286R, R375Q, V411L, and P452L were constructed using the primers summarized in **Supplementary Table 2**. A FLAG tag was added to the N-terminus *via* PCR. The recombinant FLAG-protein was expressed in 293T cells. Co-immunoprecipitation (Co-IP) was performed to isolate the FLAG-conjugated catalytic subunit using the FLAG-beads (Sigma-Aldrich, St. Louise, MO, USA) following the manufacturer’s instruction. The FLAG-tagged protein band was visualized using Coomassie blue staining and Western blot analysis with an anti-FLAG antibody (#2368T; 1:1000; Cell Signaling Technology, Danvers, MA, USA). The FLAG-tagged wildtype and mutant catalytic fragments were purified from the immunoprecipitated complexes using the 100 kDa Amicon^®^Ultra-0.5 filter (MilliporeSigma, Burlington, MA, USA).

### DNA polymerase and exonuclease assays

All oligonucleotides were purchased from Genewiz (China). The DNA polymerase and exonuclease substrates P50/T80a and P51T/T80 were prepared by annealing P50 (18) (Cy5-5’-TGGAACTTTGTACGTCCAAAATTGAATGACTTGGCCAA-3’; 50-mer) or P51T (Cy5-5’- TGGAACTTTGTACGTCCAAAATTGAATGACTTGGCCAACTACACTAAGTTT-3’; 51-mer) to T80a (5’-GGAAAACGAAACGAAGCACAGGAGCCCTGGAACTTAGTGTAGTTGGCCAAGTCATTCAATTTTGGACGTACAAAGTTCCA-3’; 80-mer) or T80 (5’-GGTTTTCTTATCGTATCACTTTTGCCCTGGAACTTAGTGTAGTTGGCCAAGTCATTCAATTTTGGACGTACAAAGTTCCA-3′; 80-mer) containing a *BsaJI* restriction site sequence (bold and underlined) for 2 min at 92°C in the presence of 150 mM NaAc.

To determine the polymerase activity, 100 nM substrate was incubated with 6.25 nM purified wildtype or mutant Polϵ, dNTPs, and PCR buffer at 30°C for 30 min. To determine the exonuclease activity, the mixture was digested with *BsaJI* (Fermentas, Waltham, MA, USA) at 60°C for 100 min. The reactions were stopped by adding the same volume of nucleic acid electrophoresis buffer. Samples were analyzed by electrophoresis in 8 M urea-12.5% polyacrylamide gels at 130 V for 55–60 min. Images were acquired using a GE Typhoon laser scanner (GE Healthcare, Munich, Germany).

### Construction of stable *POLE*-knockdown cell lines

HEK-293T cells were transfected with pGMLV-SC5 lentiviral vectors expressing negative control small hairpin RNA (shRNA) or shRNA against *POLE* (shRNA1, shRNA2, shRNA3, or shRNA4; **Supplementary Table 3**; Sangon Biotech). The knockdown efficiency was evaluated by detecting *POLE* expression using quantitative real-time PCR and Western blot analysis. The stable *POLE*-knockdown cells were selected using 1.5 μg/mL puromycin for 2 weeks and maintained in the medium containing 0.75 μg/mL puromycin.

### MTT assay

HEC-1A cells were seeded in a 96-well plate at a density of 4,000 –6,000cells/well and cultured overnight. Cells were transiently transfected with lentiviral vectors expressing wildtype *POLE* or *POLE* variants. Cells were treated with different concentrations of cisplatin (0.0001, 0.0128, 0.064, 0.32, 1.6, 8, 40, 100, 200, and 500 µmol/L) for 48–72 h. MTT (5 mg/L, Beyotime) was added to each well following the manufacturer’s instruction. After 4 h of incubation at 37 °C, the MTT-containing medium was removed, and 150 μL of dimethyl sulfoxide (Sigma-Aldrich) was added to each well. The absorbance values were measured using a microplate reader (Berthold Technologies, Oak Ridge, TN, USA) at a wavelength of 490 nm.

### Cell migration and invasion assays

Cell invasion was measured using the Transwell chamber assay. HEC-1A and AN3CA cells were transiently transfected with lentiviral vectors expressing wildtype *POLE* or *POLE* variants. A total of 2.5×10^4^ cells were loaded into a matrigel-coated upper chamber filled with serum-free medium. The lower chamber was filled with 600 μL medium containing 20% FBS. After incubation at 37°C for 24–48 h, cells remaining in the upper chamber were removed with a cotton swab. The invading cells adhering to the lower surface were fixed and stained with crystal violet (0.1%). The stained cells were counted in 3 randomly selected fields under an inverted light microscope (CKX43; Olympus, Japan) at 200× magnification.

Cell migration was measured using the wound healing assay. Cells were seeded in a 6-well plate at a density of 5 × 10^5^ cells per well and cultured overnight. Cells were transfected as above mentioned. A 10 µL micropipette tip was used to generate a 2 mm-wide scratch line in the cell monolayer. Cells were allowed to migrate for 6, 12, 24, 48, or 72 h. Images were captured under an inverted light microscope (CKX43; Olympus).

### Cell cycle analysis

POLE-knockdown HEC-1A cells were seeded in a 6-well plate at a density of 2×10^5^ per well. Cells were transiently transfected with lentiviral vectors expressing wildtype *POLE* or *POLE* variants. Cells were collected at 48 h after transfection, resuspended in 300 µL phosphate-buffered saline (PBS), and fixed with 100% ethanol overnight at -20 ˚C. After propidium iodide staining, cell cycle was analyzed using a MoFlo XDP flow cytometer (Beckman-Coulter, Brea, CA, USA).

### T7 endonuclease I mismatch cleavage assay

The abundance of mismatched base pairs was measured using T7 endonuclease I mismatch cleavage assay. Cells were fixed with 4% formaldehyde for 30 min, followed by incubation with T7 endonuclease 1 (Vezyme) at 37 °C for 45 min to cleave the mismatched base pairs. The produced 3′-OH DNA ends were stained with TUNEL using a TUNEL kit (Beyotime Biotechnology, Shanghai, China) following the manufacturer’s instruction. The samples were immediately analyzed on the flow cytometer.

### Quantitative real-time PCR (qRT-PCR)

Total RNA was isolated using TRIzol (Life Technologies, Carlsbad, CA, USA) according to the manufacturer’s instructions. cDNA was synthesized using a reverse transcription kit (Sangon Biotech) following the manufacturer’s protocol. Amplification was performed using a SYBR master mixture (Sangon Biotech) and gene-specific primers (**Supplementary Table 4**) on a qRT-PCR device (Bio-Rad, Hercules, CA, USA). β-actin was used as an internal control.

### Western blot analysis

Cells were lysed using RIPA buffer (CoWin BioSciences, China) and centrifuged at 13,000 rpm for 15 min at 4 ˚C. The supernatant was collected, and the protein concentrations were determined using a Bradford method. The protein samples were separated on a 10% SDS-PAGE gel and then transferred to a polyvinylidene fluoride membrane. After blocking with 5% skim milk at room temperature for 90 min, the membrane was incubated with primary antibodies against POLE (1:1000; Abcam, Cambridge, UK), MLH1 (1:1000; Abcam), MSH2 (1:1000; Abcam), E-cadherin (1:1000; Cell Signaling Technology, Danvers, MA, USA), N-cadherin (1:1000; Cell Signaling Technology), Slug (1:1000; Cell Signaling Technology), Snail (1:1000; Cell Signaling Technology), Cyclin A (1:1000; Santa Cruz Biotechnology, Dallas, TX, USA), Cyclin B (1:1000; Santa Cruz Biotechnology), Cyclin B1 (1:1000; Santa Cruz Biotechnology), Cyclin D1 (1:1000; Santa Cruz Biotechnology), CDK4 (1:1000; Santa Cruz Biotechnology), CDK6 (1:1000; Santa Cruz Biotechnology), P16 (1:1000; Santa Cruz Biotechnology), P21 (1:1000; Cell Signaling Technology), or β-actin (1:1000; Santa Cruz Biotechnology) overnight at 4°C. After incubation with a horseradish peroxidase-conjugated secondary antibodies (mouse or rabbit) (Sigma-Aldrich) for 1.5 h at room temperature, the protein bands were detected using a Tanon gel imaging system (Tanon Science & Technology, Shanghai, China). The results were quantitatively analyzed using a GelCap software.

### Statistical analysis

Data were expressed as the mean ± standard deviation. Statistical analysis was performed using SPSS 19.0 (IBM, Armonk, NY, USA) or Flowjo 7.6.2 (Treestar, Ashland, OR, USA). The graphs were generated using Graphpad 5.0 (GraphPad, San Diego, CA). Differences between groups were compared using one-way analysis of variance, followed by Student’s *t* test. A *P* value < 0.05 was considered statistically significant.

## Data availability statement

The original contributions presented in the study are included in the article/**Supplementary Material**. Further inquiries can be directed to the corresponding authors.

## Ethics statement

The studies involving human participants were reviewed and approved by the Ethics Committee of Fudan University Shanghai Cancer Center. Written informed consent to participate in this study was provided by the participants’ legal guardian/next of kin. Written informed consent was obtained from the individual(s), and minor(s)’ legal guardian/next of kin, for the publication of any potentially identifiable images or data included in this article.

## Author contributions

WT and ZJ designed the concept and experiments. JW and JM collected the data. RB, YR, and BS participated in the project design and manuscript discussion. GY and HW revised the manuscript. All authors contributed to the article and approved the submitted version.

## Funding

This study was supported by grants from the National Natural Science Foundation of China (No. 82002758 to WT) and the Yangfan Plan of Shanghai Science and Technology Commission (No. 22YF1404700 to ZJ).

## Conflict of interest

The authors declare that the research was conducted in the absence of any commercial or financial relationships that could be construed as a potential conflict of interest.

## Publisher’s note

All claims expressed in this article are solely those of the authors and do not necessarily represent those of their affiliated organizations, or those of the publisher, the editors and the reviewers. Any product that may be evaluated in this article, or claim that may be made by its manufacturer, is not guaranteed or endorsed by the publisher.
