# Supplementary material for: Characterization of hotspot exonuclease domain mutations in the DNA polymerase ϵ gene in endometrial cancer
Source: Front Oncol. 2022 Oct 12;12:1018034. doi: 10.3389/fonc.2022.1018034 (PMC9596989; doi:10.3389/fonc.2022.1018034)
Supplement: Supplementary file 1 [file DataSheet_1.pdf]

Supplementary Table

1 Primers for *POLE* sequencing.

Number	Forward	Reverse
**seq01**	ATGTAAATCAGAAATGCAGACACC	CACAAATACACAGTGACAACCTAG
**seq02**	CTCTATCAGCCTCAGGAAAACAAG	CAAAGACACGAGCCTCCTG
**seq03**	CACCTGTTTGCATGGAGAAATG	CAGTGAGCCAAAAGTGAGGTG
**seq04**	TAGGTGCCCCTAGTTCTTTGTC	AAAATCCAGATTCTCACGGGTTAG
**seq05**	AGGATGTGGCCCTGGC	CCAGGGGCTTTTCCTCCC
**seq06**	TCTGCGTCCGTGCCAG	CTGCTTCTTCAGGTGCTCTG
**seq07**	CTCCTCTCTGAGTGGACTGG	GATGTGGCCTTGGCATCAG
**seq08**	CTTACTGATGGGCCCACGAATG	CGAGGCAGATGAGGGAGAG
**seq09**	ACTGTGGTCTGGAGGCTG	ACTCCAGGGCACACGG
**seq10**	GAATAAGCTGAACCGAGACCTG	CTCGGATGTTCTGCTCCAC
**seq11**	TCTAAAGGGACTAAGGAAGGGAG	GAACTCGCCGACATCCAC
**seq12**	TCTCAGGATTATGTCGCAAATGAG	TACGTTAGTGTCCTCTCCTCACAC
**seq13**	GTGAGCAGTCTTTTGTCTTTTGAG	ATCTTCTGAGTGATGGTGAAGAAG
**seq14**	CTCAGATTCTAGGGTGTTTGCAG	CTCAAAAGACAAAAGACTGCTCAC

**seq15**	CAGAGGATGAGCAGGAAAATGAG	CATCTACTAACCATGAGTCCCTTC
**seq16**	GACTGCGAGGAAGGCCTC	CTCCTCCTCCCCATCTCTTTC
**seq17**	AGAACAAGGTCGATTTCACTGAAG	CAAAGCTGGCTCGGGTG
**seq18**	TCTGGCTCTCCATGACTGTG	GTCACCAGGACCAGCCAG
**seq19**	TGAAGGAGATCACCCAGTACC	ATGTCTCTGGTTCTGGGGAGTAAG
**seq20**	GACAGTGACGGATTTGGACC	GTAGAAGTGCATCACCTGGTTG
**seq21**	CATTCTCCAGTCTCACCATGTC	AATTAAGGGCAAAGGATAAGACCC
**seq22**	GTCTGTTGCTGGTTCTGGAG	GATCACGTCGAAGCTGATCC
**seq23**	CTACCAGAGGACATCTCCACATTC	CTCATGGGCCTCGTCCTC
**seq24**	TCGCAGGCCTTCGAGATG	GCAAAGAAGAGGTCGGAGC
**seq25**	CACACTCATCGCTGTTCAGTC	CAACGACAGTACTGTGCTCAC
**seq26**	GAAACTGACCTGAAGACCATCTG	TCCAAGACAGGAATTTCACTGG
**seq27**	CTGTACTCAGCAGAGCACG	TCAAGGCAAAATGGAAGAAAAGAC
**seq28**	GAGTGCTTGTCGTGATTGAATTG	CGAACTTCGAAGGTGTGTTTG
**seq29**	CAGCCGCCCATCATGTG	CAATGTGCTGCCATGGAGG
**seq30**	CACCATCCAGCCCTTGC	CACATGATGGGCGGCTG
**seq31**	GTCGTATTCTCGCCAGGTAAATC	GTTTCTTTCCTCCATACCACCC

**seq32**	TTGTAATCATTGAGAGAGGTGGAC	CTCTGGCACTGAATACTCATAGAG
**seq33**	CTTTCAGATCAGCGAGACCAG	CAGGCTAGATCATGGGAAAGC
**seq34**	CCTGAGGTCTGGGATGGG	GCCCACAGCCTGAACAG
**seq35**	GTCTGGAGTCGGCAGAGG	CTCTAACCCTCCCATCCCAG
**seq36**	GCTGCTTGTGAGAAAGTCATG	GCAGGACCATCCCGGATG
**seq37**	CGAGGCCAAGTGCTCCTG	CCTCAGAGACAGACAGTATCACAG
**seq38**	GTTACCCAGGCTGGTCTCAAACTC	GTGAGGCAGCTTTACGAGG
**seq39**	ACAAGGGTCTCTCTCTGATGG	GTCCTGTGTGTCACAAGGATC
**seq40**	TAAATAAGACACAGTGGTTAGCCC	ACAGTTTTTAGCCCCACAGC
**seq41**	TAAGAGTTAGGATCAGATTTTGGG	TTTAAGAGTAGAGAACGCAACTGG
**seq42**	CATCTCTGAGAACCGTTCCATG	CTCACCTCTCCGTGATGGG
**seq43**	ATTAGAGAGCTCTTGAAATTGCAG	TAGACTTCTGCTCCCCGTAATC
**seq44**	TCAAGGGCTTTGAGGTCAAAC	CTTCTTGCTTCATCCTTCATCCC
**seq45**	AGAGAGGATGAGGCACAGC	TACAGAGCCATACACCTCTTCC
**seq46**	TCTTCACTCTTGTTTCTCAGGAAG	CAATCCATGTCCTTTCTAAAGCAC
**seq47**	ATGCATCAGAACTTCCTTCCTTC	CGTAGGTGAGTGAGGACGG
**seq48**	CTTGCTCCTCTCTTTCTCTGAAG	CAGAGCTTCAGGACCAGAGG

**seq49**	TCTGATACCCCCTGGAGGAG	CCTTCCCTCCTTCCTTCCTG
**seq50**	CTTCTCAGGTGTGGAAAAAGAAG	GACTCATCCATTCCTCCCATAAG
**seq51**	AATGTACACTCTTCATTGTACTGG	ATACAGCACCTCCATGTTCTTG
**seq52**	CATCTCCGTCGTGCTTAGATTAC	CTGAAACTGCCCTCACAGAG
**seq53**	CAAAGTGCTGGGATTACAGGTG	ATGTGGATCTTCTTGTAGGCTTTC
**seq54**	TCTGTGCCTCCTTTTGAACTTG	ATCTTTACAGCCGTGACCATG
**seq55**	CTTTACTTCCCGTGTCAGAGTC	ACACTCACCCACCCGTTTC
**seq56**	GAACTGCACACGAGGCAAG	CTGACACGGGAAGTAAAGTCTC
**seq57**	GCTAGTGTGGGTGTGTTTCC	CCAAACACAGACTGGCTCTTC
**seq58**	CTTCCACGCCAACATCATCTTC	ACAAGAGTGGGAAGAATCTGAATC
**seq59**	TTCTCTCCTTTCACACTGAAGTTC	TTATTGAACTCCTGCTCTTGCTTG
**seq60**	CTCTTGATTTTTGATGGCCCTG	AGCTCCAGTGCATTTGGAATG
**seq61**	TTAGAATCATCCTGGCTTCTGTTC	CATCTGGATGCGTGCACAC
**seq62**	ACTTGCTTGTCTGTCCTCTTTC	CTGCAAGAGGCCTTCAGATC
**seq63**	TCTGAACTTTGGGAGAGGAATTTG	CTGCCCAGTTACTCATAGAGAAG
**seq64**	GTGGGAACGCCATTTCTTG	GGTAGTTTCCCAAGTGATACCTC
**seq65**	GGTGGGTGTTCAGGGAGG	CAGGAGCTTACTTCCCAGAAG

**seq66**	ATGGGTGAATCCACAGAAGAATAG	ACTCTCCAGCACTGAAGAATATTC
**seq67**	CAGCTGAGTTAGTTGCTCTCTTG	CTGTTAGGAAATTCATGTGAGCAG
**seq68**	CTATTTCTCTTGAACCAATGAGCG	TTTGCTCACAAGACCAAAGTTTAC
**seq69**	GGCAAGGAAGGAAATGTATTTTACC	AATGTAGACTCTGGCCTCATTTAC
**seq70**	CATTCTGACTTCCTGTATGCTACC	GACCTGAGTCTATGAAACACACTC
**seq71**	TTGTTTCTAAAGTGCTGAGTTTCC	TTTTAGCTTGTCGCAGTCAGGG
**seq72**	CTTCTTTCACTCAGGGATGATGG	TTTAGATAAGGACCACGCTATGAC
**seq73**	CAGGAGAATGGCGTGAACC	GAGTGCCGAAACTGAGGAAG
**seq74**	GATGGACGGCGGCATTTC	ATGGCACCCTCCGGAG
**seq75**	TCCCAACCCCACGGTG	CTCTGCTTCAGGGGAGAAATTTG
**seq76**	ACCCCACCTCAGGCTG	CGAGGCGCTCCAATTGG

2 Primers for plasmid construction.

Primers	Sequences (5′–3′)
H_*POLE* WT	
Primer-F1	TTGGTACCGAGCTCGGATCCGCCACCATGTCTCTGAGGAGCGGCG
Primer-R1	AATAATAGTCAACAGCGCTACCTAAGCGCTTATCTTCATCTAAAATCTCG
Primer-F2	TAGCGCTGTTGACTATTATTTTATTCAAGATGACGGAAGCAGATTTAAGG
Primer-R2	GCTGGATATCTGCAGAATTCCTAATGGCCCAGCTGTGGGT
H_ *POLE*-P286R	
Primer-F1	TTGGTACCGAGCTCGGATCCGCCACCATGTCTCTGAGGAGCGGCG
Primer-R1	GTCTCAGCATCTCGAAACTTGAGGGGCAGTTTGGTC
Primer-F2	AAGTTTCGAGATGCTGAGACAGACCAGATTATGATGA
Primer-R2	GCTGGATATCTGCAGAATTCCTAATGGCCCAGCTGTGGGT
H_ *POLE*-V411L	
Primer-F1	TTGGTACCGAGCTCGGATCCGCCACCATGTCTCTGAGGAGCGGCG
Primer-R1	TCTTGAGCCACCTGAGGCAGTCCATGTG
Primer-F2	CTGCCTCAGGTGGCTCAAGAGGGACAGTTACCTTCCTGTGG
Primer-R2	GCTGGATATCTGCAGAATTCCTAATGGCCCAGCTGTGGGT

H_ *POLE*-R375Q

Primer-F1	TTGGTACCGAGCTCGGATCCGCCACCATGTCTCTGAGGAGCGGCG
Primer-R1	AGACCGTGGACTGCTGCCTGGGCCTCCACAAATGGCCA
Primer-F2	AGGCAGCAGTCCACGGTCTG
Primer-R2	GCTGGATATCTGCAGAATTCCTAATGGCCCAGCTGTGGGT

H_ *POLE*-P452L

Primer-F1	TTGGTACCGAGCTCGGATCCGCCACCATGTCTCTGAGGAGCGGCG
Primer-R1	GCCAGAGTCTGCAGCTGCTCCGTGGCCATCCG
Primer-F2	AGCAGCTGCAGACTCTGGCCACGTATTCTGTG
Primer-R2	GCTGGATATCTGCAGAATTCCTAATGGCCCAGCTGTGGGT

3 shRNA sequences against *POLE*.

shRNA	Sequence (5′–3′)
shRNA1	GCAGTGCAGTGGATTACTACT
shRNA2	GCTGAGACAGACCAGATTATG
shRNA3	GCTCTTCCCTTCAAGACTTTG
shRNA4	GGGAATTTCTTCTCTGGATGG

4 Quantitative real-time PCR primers

Gene	Sequence (5′–3′)
GAPDH-F	GTCTCCTCTGACTTCAACAGCG
GAPDH-R	ACCACCCTGTTGCTGTAGCCAA
POLE-F	GGTGGGTGAAGAGGGACAGTTA
POLE-R	AGGTAGTAAGTGGCGACAGCATC
MLH1-F	CTTCGTGGGCTGTGTGAATCC
MLH1-R	CTAAGGCAAGCATGGCAAGGTC
MSH2-F	GATCATCACTTCGGTGGTCAGT
MLH2-R	GCCATTACAGGTGCATCAACAG
