# Supplementary figures and images for: Characterization of hotspot exonuclease domain mutations in the DNA polymerase ϵ gene in endometrial cancer
Source: Front Oncol. 2022 Oct 12;12:1018034. doi: 10.3389/fonc.2022.1018034 (PMC9596989; doi:10.3389/fonc.2022.1018034)

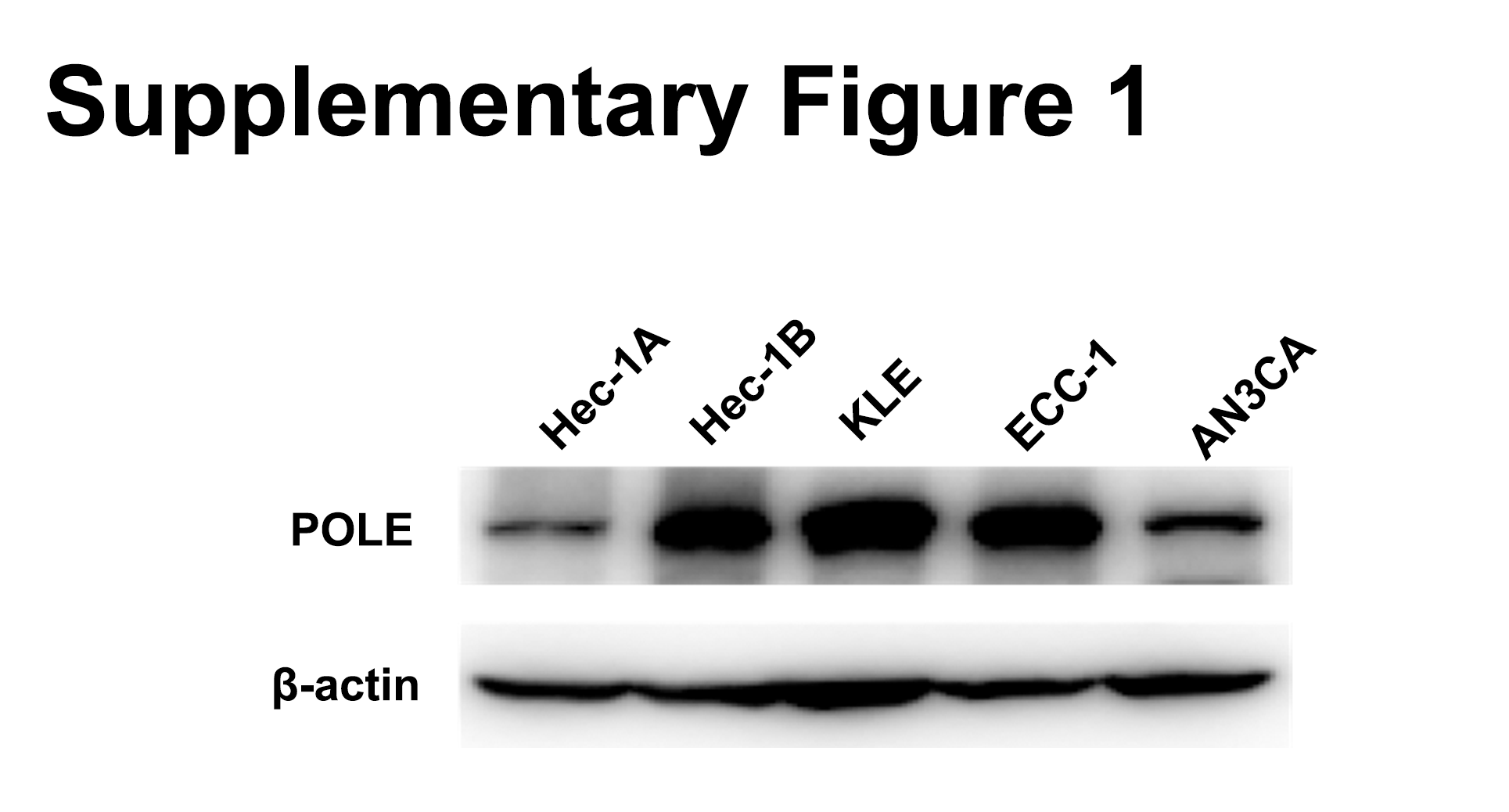

Supplement: Supplementary file 2 [file Image_1.tif]

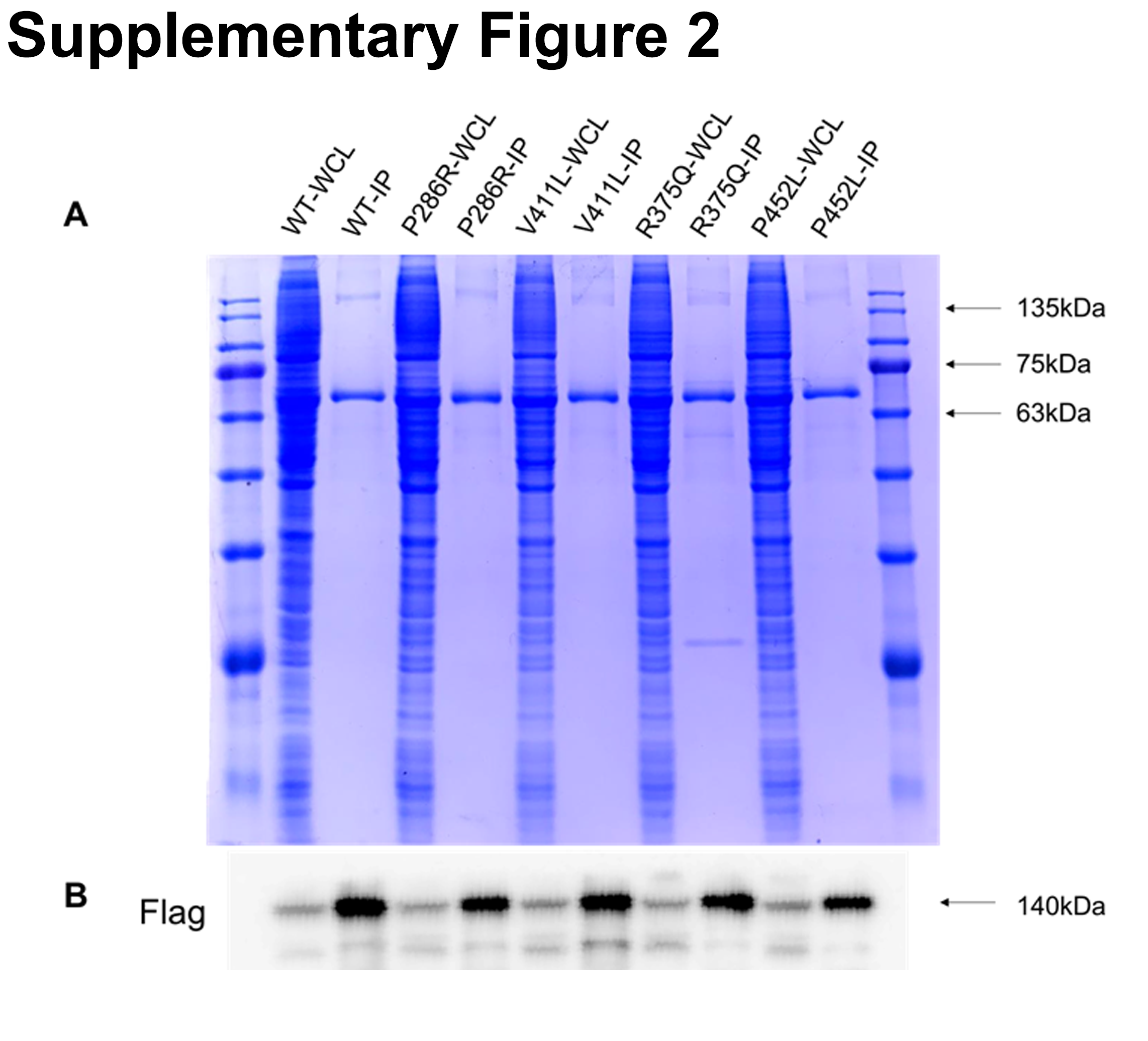

Supplement: Supplementary file 3 [file Image_2.tif]
